# Outcomes of children with idiopathic steroid resistant nephrotic syndrome: a single centre observational study

**DOI:** 10.1590/2175-8239-JBN-2022-0073en

**Published:** 2022-09-30

**Authors:** Om P. Mishra, Minketan Sidar, Vineeta V. Batra, Rajniti Prasad, Ankur Singh, Abhishek Abhinay, Akash Mishra, Ashish K. Yadav

**Affiliations:** 1 Banaras Hindu University, Institute of Medical Sciences, Department of Pediatrics, Division of Pediatric Nephrology, Varanasi, India.; 2 G. B. Pant Institute of Post Graduate Medical Education & Research, Department of Pathology, New Delhi, India.; 3 Jawahar Lal Institute of Postgraduate Medical Education and Research, Department of Biostatistics, Puducherry, India.; 4 Banaras Hindu University, Institute of Medical Sciences, Center of Biostatistics, Varanasi, India.

**Keywords:** Nephrotic Syndrome, Steroid resistant, Remission, Kidney Function survival, Síndrome Nefrótica, Córtico-resistente, Remissão, Sobrevida da Função Renal

## Abstract

**Introduction:**

Idiopathic steroid resistant nephrotic syndrome (SRNS) has variable outcomes in children. The primary objective of the present study was to assess the cumulative remission rate and the secondary objectives were to assess factors affecting the remission status, kidney function survival, and adverse effects of medications.

**Methods:**

One hundred fourteen patients with SRNS were included. Calcineurin inhibitor-based treatment protocol along with prednisolone and angiotensin-converting enzyme inhibitor were used, and patients were followed over 5 years.

**Results:**

Median age was 4.5 years; 53.5% of cases were between 1 to 5 years of age. Sixty-two patients (54.4%) were at initial stage and 52 (45.6%) were at a late SRNS stage. Median eGFRcr was 83.5 mL/min/1.73m^2^ at presentation. Of the 110 patients, 63 (57.3%) achieved remission [complete remission 30 (27.3%), partial remission 33 (30%)], and 47 (42.7%) had no remission. Kidney function survival was 87.3% and 14 cases (12.7%) had progression to CKD (G3-8, G4-3, G5-1, and G5D-2). Median duration of follow up was 36 months (IQR 24, 60). Age of onset, cyclosporine/tacrolimus, eGFRcr, and histopathology (MCD/FSGS) did not affect remission. Similarly, remission status in addition to age of onset, drug protocol, and histopathology did not significantly affect kidney function during a period of 5 years. Hypertension, cushingoid facies, short stature, cataract, and obesity were observed in 37.7, 29.8, 25.5, 17.5, and 0.7% of cases, respectively.

**Conclusion:**

About half of the cases achieved remission. Age of onset of disease, cyclosporine/tacrolimus use, and histopathological lesion neither affected remission status nor short-term kidney function survival in SRNS.

## RESUMO

**Introdução:**

A síndrome nefrótica idiopática córtico-resistente (SNICR) apresenta desfechos variáveis em crianças. O objetivo principal deste estudo foi avaliar a taxa de remissão cumulativa. Os objetivos secundários foram avaliar fatores que afetam status de remissão, sobrevida da função renal e efeitos adversos de medicamentos.

**Métodos:**

Foram incluídos 114 pacientes com SNCR. Utilizou-se protocolo de tratamento baseado em inibidores de calcineurina juntamente com prednisolona e inibidor da enzima conversora de angiotensina. Os pacientes foram acompanhados durante 5 anos.

**Resultados:**

A idade mediana foi 4,5 anos; 53,5% dos casos tinham entre 1 e 5 anos. 62 pacientes (54,4%) estavam em estágio inicial; 52 (45,6%) em estágio tardio da SNCR. A TFGecr mediana foi 83,5 mL/min/1,73 m^2^ na apresentação. Dos 110 pacientes, 63 (57,3%) alcançaram remissão [remissão completa 30 (27,3%), remissão parcial 33 (30%)], e 47 (42,7%) não apresentaram remissão. A sobrevida da função renal foi 87,3%; 14 casos (12,7%) progrediram para DRC (G3-8, G4-3, G5-1, G5D-2). A duração mediana do acompanhamento foi 36 meses (IIQ 24, 60). Idade no início, ciclosporina/tacrolimus, TFGecr e histopatologia (DLM/GESF) não afetaram a remissão. Igualmente, status de remissão, além da idade no início, protocolo de medicamentos e histopatologia não afetaram significativamente a função renal por 5 anos. Observou-se hipertensão, fácies cushingoide, baixa estatura, catarata e obesidade em 37,7; 29,8; 25,5; 17,5; e 0,7% dos casos, respectivamente.

**Conclusão:**

Aproximadamente metade dos casos alcançou remissão. Idade no início, uso de ciclosporina/tacrolimus e lesão histopatológica não afetaram o status de remissão nem a sobrevida da função renal a curto prazo na SNICR.

## Introduction

Nephrotic syndrome is the most common glomerular disorder in childhood, with an annual incidence of approximately 2 to 7 per 100,000 children below 16 years of age^1^. In Asia, a higher incidence of 9–16 per 100,000 children per year has been reported^2^. About 85–90% of children with nephrotic syndrome are idiopathic, with a favourable response to corticosteroid, but approximately 10–15% remain initially unresponsive or later develop steroid-resistance^3^. Steroid resistant nephrotic syndrome (SRNS) patients show absence of remission despite therapy with daily prednisolone at a dose of 2 mg/kg or 60 mg/m^2^ for 4 weeks^4^, which has been recently modified to 6 weeks^5^. SRNS has been associated with unfavourable prognosis, with 36–50% of patients progressing to end stage kidney disease (ESKD) within 10 years^6,7^.

Histological subtypes of SRNS include mainly focal and segmental glomerulosclerosis (FSGS), minimal change disease (MCD), and diffuse mesangial proliferation (DMP)^8,9^. Mutations in podocyte-associated genes can be found in 10–30% of non-familial SRNS^10–12^. More than 50 genes have been identified to date and most of these are localized in the podocyte or in the slit diaphragm, thereby confirming the importance of podocyte dysfunction in the pathogenesis^5,13^. Common mutations registered in the PodoNet Registry are NPHS2, WT1, and NPHS1, which included children with steroid-resistant and congenital nephrotic syndrome^14^. However, mutations in the NPHS2 gene represent 20 to 30% of sporadic SRNS^15^.

Treatment of patients with non-genetic forms of SRNS usually includes inhibitors of the renin-angiotensin-aldosterone system and calcineurin inhibitors (CNI). Complete or partial remission can be achieved in 50–70% of non-genetic SRNS cases^3,16^. In an analysis of immunosuppressive therapy given in a large cohort of SRNS patients in the 1^st^ year after diagnosis, 62% of the children were treated with a single immunosuppressant, 28% with two immunosuppressants, and 10% with three or more different immunosuppressive drugs in combinations. Only 41% of patients responded to immunosuppressive drug therapy with proteinuria reduction, the highest remission achieved with CNI-based treatment protocol^17^. Further, SRNS patients can also show a multidrug-resistant phenotype who also do not respond to immunosuppressive therapies such as CNI, prednisolone, and rituximab^18^.

Preserved kidney function has been reported to be 75% at 5 years, 58% at 10 years, and 53% at 15 years^6^. Inaba et al.^19^ observed that kidney function survival rate was significantly different among the four different sub-groups based on different combinations of initial histopathological lesions (FSGS vs MCD/DMP) and immunosuppressants given for SRNS.

We present the analysis of data from our SRNS patients. The primary objective was to assess the cumulative remission rate (complete, partial, and no remission) and secondary objectives were to assess factors affecting remission status and kidney function survival, and also to record side- effects of immunosuppressive medications.

## Materials and Methods

The study was conducted in the Division of Pediatric Nephrology at a tertiary care centre of a teaching hospital. The study was based on review of data (September 2009 to June 2021) as a longitudinal observation. Patients of sporadic idiopathic SRNS, aged 3 months to 18 years were included. The children who did not achieve remission with daily oral prednisolone at a dose of 2 mg/kg/day or 60 mg/m^2^/day for 4 weeks were categorized as SRNS^4^. Patients with congenital or syndromic forms, positive family history of nephrotic syndrome, secondary etiologies such as systemic lupus erythematosus, drug induced nephropathy, IgA nephropathy, HIV and hepatitis B infection, and those who did not complete the treatment protocol were excluded.

The medical records of each study subject were reviewed with respect to history, physical examination, and investigations. All patients had their weight, height, body mass index, and blood pressure (BP) recorded. Office BP was measured and hypertension was defined as systolic and/or diastolic BP ≥ 95th percentile for age, gender, and height recorded on 3 or more different occasions^20^. Estimated glomerular filtration rate creatinine (eGFRcr) was calculated using the modified Schwartz formula^21^. Grading of chronic kidney disease (CKD) was done as per the Kidney Disease Outcome Quality Initiative Guidelines^22^.

Investigations included hemoglobin, total and differential leukocyte counts, platelet counts, serum total protein, albumin, cholesterol, urea, creatinine, sodium, potassium, random blood sugar, and T3, T4 and TSH. Screening for HIV, hepatitis B, and tuberculosis (chest X-ray and Mantoux test) were done in all patients. Serum C3, C4, ANA, and anti-ds DNA and ultrasonography of kidney, ureter, and bladder were done, whenever indicated.

Urine was examined for the presence of pus cells, red blood cells (microscopic hematuria was defined as the presence of ≥ 5 RBCs per high power field in a centrifuged fresh urine specimen) and casts. Urine protein testing was done by Dipstick and urinary protein/ creatinine ratio (Upr/cr) expressed as mg/mg was measured in a spot sample.

## Treatment Protocol

Patients were treated with prednisolone (2 mg/kg/day or 60 mg/m^2^/day in single or two doses) as per Indian Society of Pediatric Nephrology Guidelines^4^. In those who did not achieve remission, i.e., who had proteinuria ≥++ by heat precipitation method/Dipstick or Upr/cr of >2 mg/mg for 3 consecutive days over a 4-week period, a kidney biopsy was performed. The histopathological tissues were examined by light and immunofluorescent microscopy and, where indicated, electron microscopy by the same nephropathologist. The study protocol was approved by the Institute’s Ethical Committee.

The SRNS patients were treated with cyclophosphamide infusion (500 mg/m^2^, monthly, 6 doses) or calcineurin inhibitor (cyclosporine 4–6 mg/kg/day with trough level of 80–120 ng/mL or tacrolimus 0.1–0.2 mg/kg/day with trough level of 5–9 ng/mL, each in two doses) along with prednisolone in alternate days (1–1.5 mg/kg in gradual tapering doses for the first 6 months) and ramipril 6 mg/m^2^/day was given for 2 years.

## Follow-Up

Patients were followed-up at 6 months, 12, 24, 36, 48, and 60 months to assess remission status (complete, partial, or no remission), evaluation of clinical profile, kidney function, adverse effects of medications, progression to CKD, and mortality. Serum creatinine was measured at baseline and subsequently and eGFRcr was calculated. Clinical data were recorded from the diagnosis of SRNS up to their last follow-up.

Drugs were changed for the cases who did not achieve remission by 6 months or developed deranged kidney function following cyclosporine or tacrolimus therapy. In children on CNI-based regimen, drugs were stopped at 2 years of treatment completion and a new kidney biopsy was not performed. Mycophenolate mofetil (1000–12000 mg/m^2^/day in two doses) was given along with prednisolone in alternate days in cases that needed change of therapy. If the patient was non-responsive to this therapy, two doses at two weeks interval of rituximab infusion (375 mg/m^2^/dose) was administered.

Complete remission was defined as no or traces of urine protein by urine dipstick or protein/creatinine ratio <0.2 mg/mg for 3 consecutive days. Partial remission was defined as urine 1+ or more by dipstick or Upr/cr between 0.2 and 2.0 mg/mg, and no remission as urine albumin >++/+++ by dipstick test or Upr/cr of >2.0 mg/mg.

## Statistical Analysis

Data were analyzed using SPSS version 23.0 software. Values were expressed as number and percentage for categorical variables. Quantitative data with Gaussian distribution are expressed as mean ± SD and data of non-Gaussian distribution are shown as median and IQR (interquartile range). Chi-square test was applied for comparison of data in proportions. Student’s t-test and Mann-Whitney U-tests were applied for comparison between two groups with Gaussian and non- Gaussian distributions, respectively. Kaplan-Meier analysis and log-rank tests were used for cumulative remission status in relation to age of onset of disease, initial immunosuppressive medications, histopathology and initial eGFRcr, and kidney function survival according to age of onset, immunosuppressive medications, histopathology, and remission status (complete + partial vs no remission). Cox regression analyses were performed to assess risk factors for non-responsiveness and progression to CKD. A p value of <0.05 was considered significant.

## Results

**Figure 1.**
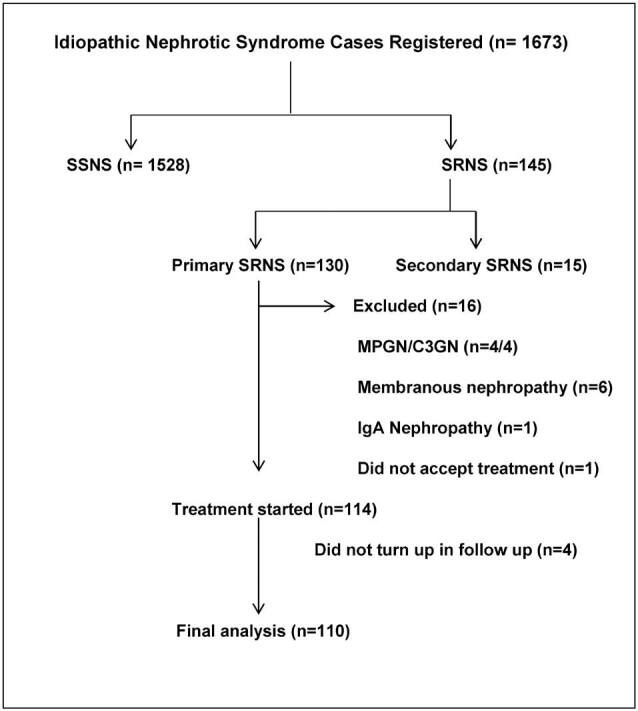
Study flow of participants.

A total of 1673 patients of idiopathic nephrotic syndrome were included, of which 1528 were of steroid-sensitive nephrotic syndrome (SSNS). The remaining 145 (8.7%) cases were of SRNS, 130 (114 retrospective + 16 prospective) of which were idiopathic SRNS (7.8%) and 15 (0.9%) were secondary SRNS, including 9 lupus nephritis, 3 crescentic glomerulonephritis, and one case of pauci-immune glomerulonephritis, hepatitis B nephropathy, and Sjogren’s syndrome each. Of 130 idiopathic SRNS, 4 patients had membranoproliferative glomerulonephritis, 4 had C3 glomerulonephritis, 6 had membranous nephropathy, and 1 had IgA nephropathy, while one patient with FSGS did not accept treatment. Treatment was started in the remaining 114 SRNS patients. Furthermore, 4 patients did not show up for follow up after treatment initiation. Thus, 110 patients were finally included in the analysis (Figure 1).

Clinical parameters of the114 SRNS cases at initial presentation are shown in Table 1. About half of the cases (53.5%) were in the age group of 1 to 5 years, with median of 4.5 years (IQR 2, 8). There were 74 males (64.9%) and 40 females (35.1%). Sixty-two patients (54.4%) were of initial SRNS and 52 (45.6%) were late SRNS. Median eGFRcr was 83.5 mL/min/1.73 m^2^ (IQR 65.6, 102). Edema was present in 97.4% and hypertension and microscopic hematuria were present in 37.7% of cases, each. Hypothyroidism was found in 11 (9.6%) cases (5 subclinical and 6 overt). Median urine protein/creatinine (Upr/cr) ratio was 15.1 mg/mg. Mean serum albumin and cholesterol were 1.7 g/dL and 410 mg/dL, respectively. Histopathological subtypes were MCD in 63 (55.3%), FSGS in 48 (42.1%), and mesangial proliferation in 3 (2.6%) patients. Genetic mutations could be tested by next generation sequencing in only 39 cases, and 4 patients (10.3%) had mutations. Two heterozygous variants in the NPHS1 gene were detected in one patient, which was categorized as pathogenic; the patient had FSGS and achieved complete remission. Another pathogenic variant in the NPHS2 gene was found and the case had FSGS histology and did not attain remission. A novel likely pathogenic heterozygous variant in the INF2 gene was detected and the patient had FSGS histology and also did not achieve remission. Two heterozygous variants of unknown significance in CRB2 gene were also detected in a patient who had MCD at histology analysis and attained complete remission.

**Table 1.** Clinical parameters of SRNS (N = 114)

Parameters	Findings
Age groups 6 m–1y 1–5 y 6–12 y 13–18 y	3 (2.6%) 61 (53.5%) 44 (38.6%) 6 (5.3%)
Gender Male Female	74 (64.9%) 40 (35.1%)
Category Initial Late	62 (54.4%) 52 (45.6%)
Height (cm)	106.3 ± 24.3
Weight (kg)	19.2(14, 27.6)^a^
Body mass index (kg/m^2^)	18.2 ± 3.6
Systolic BP (mm Hg)	105.1 + 14.9
Diastolic BP (mm Hg)	68.6 + 11.7
Estimated glomerular filtration rate (eGFRcr) (mL/min/1.73 m^2^)	83.5 (65.6,102)^a^
Edema	111 (97.4%)
Hypertension	43 (37.7%)
Hematuria	43 (37.7%)
Hypothyroidism	11 (9.6%)
History of consanguinity	2 (1.7%)
Genetic mutations (n = 39) Present Absent	4 (10.3%) 35 (89.7%)
Histopathology Minimal change disease Focal segmental glomerulosclerosis Diffuse mesangial proliferation	63 (55.3%) 48 (42.1%) 03 (2.6%)
Hemoglobin (g/dL)	11.1 ± 2.1
Total leucocyte counts (ṡ10^3^/mm^3^)	12.4 (9087,16167)^a^
Absolute neutrophil counts (ṡ10^3^/mm^3^)	6.0 ± 1.5
Absolute lymphocyte counts (ṡ10^3^/mm^3^)	3.2 ± 1.3
Absolute platelets count (lakh per mm^3^)	4.4 ± 1.8
Serum urea (mg/dL)	29.6 (20.9,43.3)^a^
Serum creatinine (mg/dL)	0.5 (0.4,0.5)^a^
Serum total protein (g/dL)	4.3 + 0.9
Serum albumin (g/dL)	1.7 ± 0.4
Serum cholesterol (mg/dL)	410 ± 145
Urine protein/creatinine ratio (mg/mg)	15.1 (9.1, 23.2)^a^

n: number of cases, a: data as median and interquartile range.

## Response to Immunosuppressive Therapy

Immunosuppressive therapy was given to all the 114 cases of SRNS; tacrolimus in 64 (56.1%), cyclosporine in 46 (40.4%), and intravenous cyclophosphamide in 4 (3.5%) patients. Out of 110 CNI-based treatment, 63 (57.3%) patients achieved remission [complete remission in 30 (27.3%), partial remission in 33 (30%)] and 47 (42.7%) had no remission. Alternate medications such as mycophenolate mofetil and rituximab were also used for patients who developed drug-related toxicity or in those whom CNI treatment protocol was completed after 2 years. Overall remission was achieved in 40.4% of such patients. Variables affecting remission status are presented in Table 2. Age of onset of disease, serum protein, Upr/cr, histopathology, and time to remission did not differ significantly between the two groups, except a significantly lower serum albumin in patients with no remission.

**Table 2.** Patient characteristics by response to immunosuppressive therapy

Parameters	Remission (n = 63)	No remission (n = 47)	P
Age (months) <60 >60	37 (58.7%) 26 (41.3%)	25 (53.2%) 22 (46.8%)	0.681^a^
Total serum protein (g/dL)	4.3 ± 0.9	3.0 ± 0.6	0.702^b^
Serum albumin (g/dL)	2.9 (2.6, 3.3)	2.1 (2.0, 2.5)	0.003^c^
Upr/cr (mg/mg)	14.8 (9.6, 22.4)	16.7 (8.9, 25.7)	0.370^c^
Histopathology MCD FSGS	36 (57.1%) 27 (57.4%)	27 (42.9%) 20 (42.6%)	0.596^a^
eGFRcr (mL/min/1.73 m^2^)	89.2 ± 27.6	88.5 ± 32.3	0.538^b^
Time to remission (months)	3.2 ± 0.5	3.5 ± 0.4	0.550^b^

n: number of cases, eGFRcr: estimated glomerular filtration rate creatinine, Upr/cr: urine protein/creatinine, MCD: minimal change disease, FSGS: focal segmental glomerulosclerosis. a: Chi-square test; b: Student’s t-test; c: Mann-Whitney U-test.

Thirteen cases (11.8%) had eGFRcr <60 mL/min/1.73 m^2^ (G3) at presentation, of whom 9 recovered, while 10 additional patients progressed to CKD during follow-up over 5 years. A total of 14 children (12.7%) progressed to CKD (G3-8, G4-3, G5-1, and G5D-2). Thus, ESKD-free survival was 87.3%. Univariate analysis of factors affecting kidney function survival is presented in Table 3; median duration of follow up was 36 months (IQR 24, 60). Age of onset of disease, gender distribution, remission status, histopathology, drug treatment, hypertension, initial/late SRNS, Upr/cr, and serum albumin did not differ significantly between the cases who progressed to CKD and those who had normal kidney function survival at their last follow up.

**Table 3.** Analysis of variables in relation to kidney function status during follow-up

Parameters	eGFRcr ≥ 60 mL/min/1.73 m^2^ (n = 96)	eGFRcr < 60 mL/min/1.73 m^2^ (n = 14)	P
Age at onset <5 years >5 years	52 (54.2%) 44 (45.8%)	10 (71.4%) 04 (28.6%)	0.262^a^
Gender Male Female	63 (65.6%) 33 (34.4%)	10 (71.4%) 04 (28.6%)	0.770^a^
Remission (n = 110) Complete remission No remission	58 (60.4%) 38 (39.6%)	05 (35.7%) 09 (64.3%)	0.116^a^
Histopathology MCD FSFS Mesangial proliferation	54 (56.3%) 39 (40.6%) 03 (3.1%)	06 (42.8%) 08 (57.2%) 00 (0.0%)	0.375^a^
Treatment (n = 110) Cyclosporine Tacrolimus Cyclophosphamide	41 (42.7%) 52 (54.2%) 03 (3.1%)	05 (35.7%) 09 (64.3%) 00 (0.0%)	0.554^a^
Hypertension Yes No	35 (36.5%) 61 (63.5%)	06 (42.9%) 08 (57.1%)	0.427^a^
SRNS Initial SRNS Late SRNS	50 (52%) 46 (48%)	08 (57.1%) 06 (42.9%)	0.475^a^
Upr/cr (mg/mg)	9.0 (2,12.6)	11.9 (7.5,18.5)	0.465^b^
Serum albumin (g/dL)	3.0 ± 1.9	2.2 ± 0.59	0.212^c^

n: number of cases, eGFRcr: estimated glomerular filtration rate creatinine, MCD: minimal change disease, FSGS: focal segmental glomerulosclerosis. a: Chi-square test; b: Mann-Whitney U-test; c: Student’s t-test.

**Figure 2.**
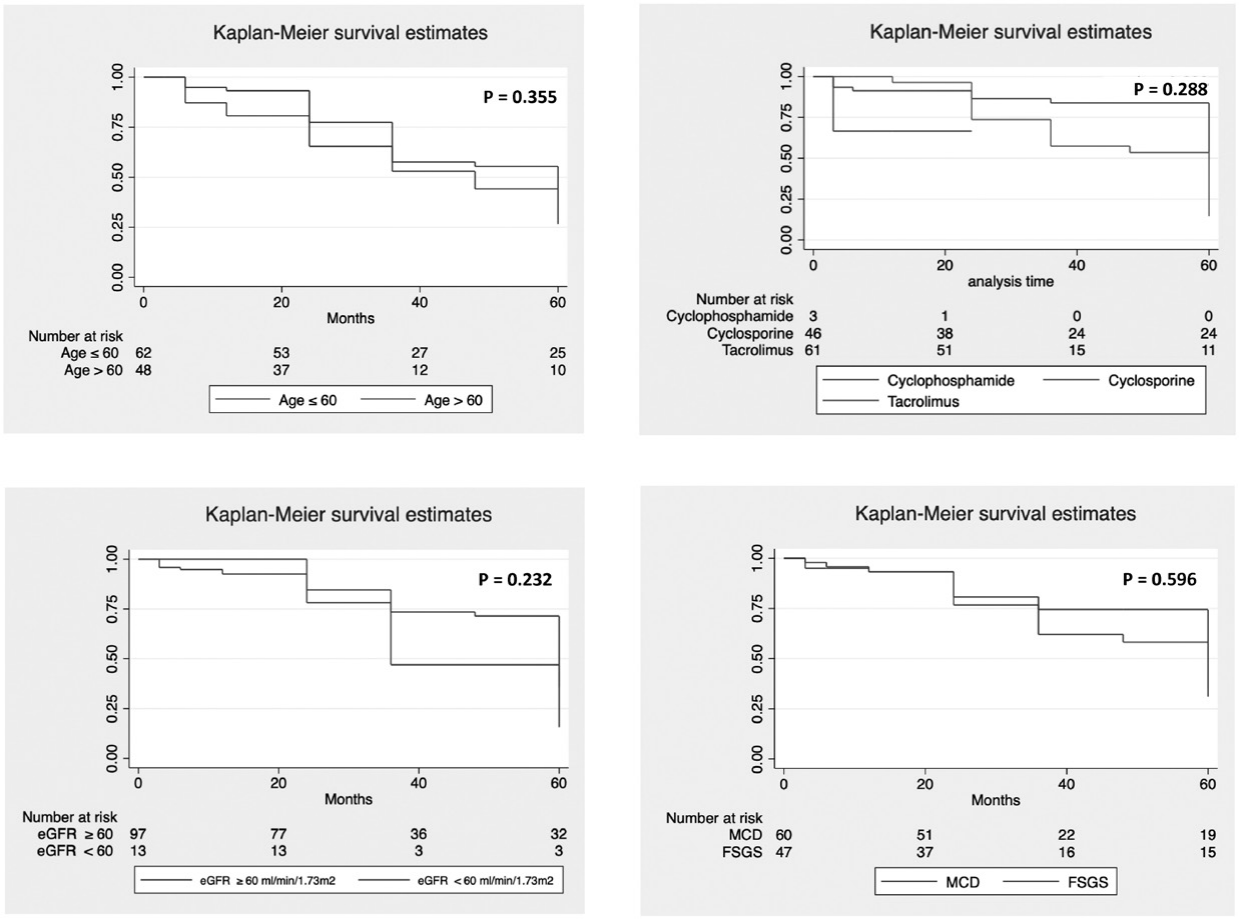
Remission status in relation to age of onset of disease, drug treatment, eGFRcr, and histopathology.

Kaplan Meier analysis was conducted for the factors affecting the remission status and results are shown in Figure 2. Patients with age of onset ≤ 60 months, those who received cyclosporine, cases with eGFRcr ≥60 mL/min/1.73 m^2^ and histopathological lesion of MCD achieved higher remission rates, but the differences were not statistically significant in comparison to the age of onset >60 months, treatment with tacrolimus, eGFRcr <60 mL/min/1.73 m^2^, and FSGS histopathology, respectively. Cox-regression analysis also did not show significant parameters predictive of non-remission (Table 4).

**Table 4.** Hazard ratio for non-remission status

Parameters	Hazard ratio	95% CI	P
Age of onset >60 months (ref. ≤ 60 months)	1.4	0.7–2.6	0.35
Drug Tacrolimus (ref. cyclosporine)	0.5	0.3–1.0	0.06
Histopathology FSGS (ref. MCD)	1.2	0.6–2.1	0.64
eGFRcr < 60 mL/min/1.73 m^2^ (ref. ≥ 60 mL/min/1.73 m^2^	0.7	0.3–1.5	0.32

eGFRcr: estimated glomerular filtration rate creatinine, MCD: minimal change disease, FSGS: focal segmental glomerulosclerosis.

**Figure 3.**
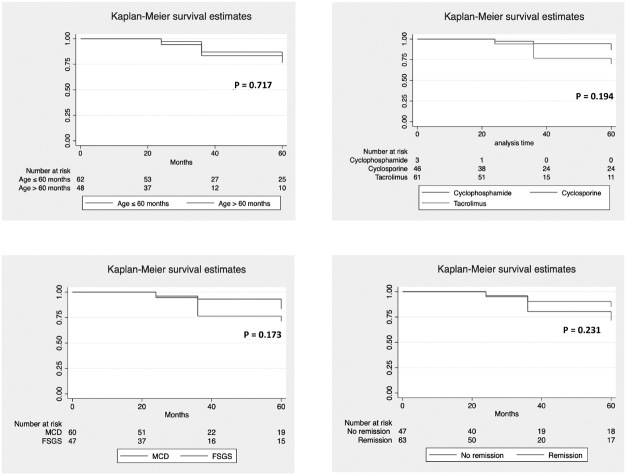
Kidney function survival in relation to age of onset of disease, drug treatment, histopathology, and remission status.

Similarly, variables affecting kidney function survival were also analyzed by Kaplan Meier analysis and it was found that none of the parameters such as age of onset of disease, drug treatment protocol, histopathology, and remission status significantly affected kidney function during the follow up period of five years (Figure 3). Cox-regression analysis revealed no variable significantly associated with progression to CKD (Table 5).

**Table 5.** Hazard ratio for kidney function (progression to CKD)

Parameters	Hazard ratio	95% CI	P
Age of onset >60 months (ref. ≤ 60 months)	0.7	0.2–1.9	0.40
Drug Tacrolimus (ref. cyclosporine)	0.4	0.1–1.3	0.12
Histopathology FSGS (ref. MCD)	1.9	0.6–6.0	0.30
Remission status No remission (ref. remission)	0.7	0.2–2.3	0.54

MCD: minimal change disease, FSGS: focal segmental glomerulosclerosis, CKD: chronic kidney disease.

The adverse effects observed included hypertension in 37.7%, cushingoid appearance in 29.8%, short stature in 25.5%, cataract in 17.5%, and obesity in 0.7% of patients. Eight patients (7.3%) died during the study period, of which 4 from sepsis (3.6%), 3 (2.7%) had CKD, and one child (0.9%) from concomitant COVID-19 infection.

## Discussion

In the present study, 7.8% of the children had sporadic idiopathic SRNS. Other authors have reported relatively higher incidence (12.7%–15%) in their series^23,24^. Nearly half (53.5%) of our cases had age of onset of disease between 1–5 years (median 4.5 years). Trautmann et al.^17^ reported similar incidence of SRNS between 1–5 years, and Mekahli et al.^6^ found similar median age in their study subjects. In contrast, Inaba et al.^19^ observed lower median age (3.2 years). Ali et al.^25^ reported that 29.2% of the sample had age of onset of SRNS between 1–5 years of age and 46.5% between 5–10 years of age. The male:female ratio was 1.8:1, which is in accordance with the reports of other authors (1.4–1.9:1)^6,19^.

Median eGFRcr was 83.5 mL/min/1.73 m^2^ at presentation. Progression to CKD occurred in 12.7% of cases at the end of the 5-year follow-up. Patients with SRNS can progress to CKD and this is due to underlying histology, such as FSGS, and non-responsiveness to immunosuppressive therapy^17,19^. We found initial SRNS in 54.4% and late SRNS in 45.6% of cases, and similar proportions (54.7% v/s 45.3%) were reported by Kim et al.^23^ Other authors found relatively higher proportion of initial SRNS (58–59%)^6,24^. Hematuria and hypertension were each present in 37.7% of all patients in the present study. Ali et al.^24^ reported hypertension in 48% and hematuria in 57% of cases. However, Mekahli et al.^6^ observed hypertension only in 8% cases and microscopic hematuria in a similar proportion as our study (57%). Thus, these two clinical features are consistently associated with patients with SRNS and this is because of underlying histopathological lesions like FSGS, DMP, and other forms of glomerulonephritis in these patients.

Hypothyroidism was detected in 9.6% of patients. Hypothyroidism was found in 20–26.2% of SRNS patients and the majority of the cases had sub-clinical hypothyroidism^25,26^. Loss of proteins in urine, such as thyroid binding protein, pre-albumin, and albumin, result in decreased level of serum protein, thyroglobulin, and T3 levels. However, most SRNS patients are euthyroid because the thyroid gland is able to compensate urinary losses of hormones. However, children with hypothyroidism may need thyroxine supplementation to achieve remission^25^.

Histopathological findings revealed MCD in 55.3%, FSGS in 42.1%, and mesangial proliferation in 2.6% of patients. MCD, the predominant histopathological lesion in SRNS, has been previously reported to range between 45 to 57.1%^6,8,19^. In contrast, other authors reported FSGS as the predominant (56–59%) histopathological lesion^9,17^. We observed a low incidence (2.6%) of mesangial proliferation compared to 11–13% reported in other series^6,17,19^. The histopathological lesion in SRNS is a significant factor for response to immunosuppressive therapy. Thus, there is heterogeneity in histopathological lesions in patient with SRNS, which may vary from region to region and can also affect long-term outcomes.

## Response to Immunosuppressive Therapy

Overall cumulative remission was found to be 57.3% (complete 27.3%, partial 30%) and 42.7% of cases had no remission. Previously, different rates of complete remission (30–45.2%), partial remission (13–19.3%), and total remission (49 to 83%) have been reported by different authors depending on the selection criteria^6,17,19,27^. As such, clinical, hematological and biochemical parameters were comparable in patients with MCD and FSGS in our study. In addition, remission status in relation to histopathology (MCD/FSGS) was also similar. The patients with no remission had significantly lower serum albumin and continued proteinuria. Alternate medications resulted in remission in 40.4% of such patients. Therefore, additional drugs as mycophenolate mofetil and rituximab are also recommended as second line drugs in the treatment of SRNS^5,13^.

Genetic mutations were found in 10.3% of patients. *NPHS1*, *NPHS2*, *CRB2*, and *INF2* mutations were detected in one case each. Nephrotic syndrome caused by *NPHS1* gene mutations show resistance to steroid therapy^14,15^. As such, a monogenic cause in SRNS has been reported in 10–30% of SRNS cases^11^. One of the largest cohorts found that a disease-causing mutation in monogenic *SRNS* gene was detected in 29.5% of the family^10^. Mutations in the *INF2* are the most common cause of autosomal dominant FSGS. Barua et al.^28^ reported that INF2 was the cause of autosomal dominant FSGS in 11 of 93 families screened. Ebarasi et al.^29^ identified a recessive mutation in *CRB2* in four different families affected by SRNS. However, Indian studies observed a very low cumulative frequency of 3.7% of mutations in *SRNS* ^30,31,32^. Patients with NPHS1 and CRB2 mutations achieved remission, while cases of NPHS2 and INF2 mutations did not respond to immunosuppression in the present study. Trautmann et al.^14^ reported that genetic abnormalities were found in 22% of patients with FSGS and in 12% of patients with MCD. Therefore, genetic mutations are more common than MCD in FSGS histology. Both group of patients show steroid unresponsiveness. However, long-term preservation of kidney function is better in MCD than in FSGS in patients with SRNS^17^. Thus, presence of genetic mutations definitely affects the outcome, but its incidence is low in the Indian population.

Risk factors for overall remission status were evaluated in this cohort. Age of onset of disease (≤ 60 months), use of cyclosporine, eGFRcr (>60 mL/min/1.73 m^2^), and MCD histopathology were related with better cumulative remission status compared to their corresponding group, but the differences were statistically non-significant. Further, Cox regression analysis did not show any association of these factors with remission. It appears that these demographic factors do not influence the overall cumulative remission in SRNS patients. Inaba et al.^19^ reported that patients with FSGS (cyclosporine), FSGS (cyclophosphamide), MC/DMP (cyclosporine), and MC/DMP (cyclophosphamide) groups had remissions of 10, 27.3, 24.1, and 23.1%, respectively, but without significant differences between the groups.

Further data were analyzed between patients having normal kidney function (eGFRcr (≥60 mL/min/1.73 m^2^) and those who progressed to CKD (eGFRcr <60 mL/min/1.73 m^2^), but none of the parameters, including age of onset of disease, gender, remission status, histopathology, types of immunosuppressive drugs, and SRNS category (initial/late), differed significantly between the two groups. In Kaplan Meier survival analysis, the factors age of onset, histopathology, type of drugs, and remission status did not influence kidney function survival. Cox regression analysis also did not show any significant predictor for progress to CKD over a period of five years. Inaba et al.^19^ demonstrated that significant risk factors for ESKD were age at diagnosis ≥11years, FSGS in initial histology, and cyclophosphamide as first immunosuppressive agent. Mekahli et al.^6^ reported kidney survival rates of 75, 58, and 53% after five, ten, and fifteen years, respectively; age of onset of nephrotic syndrome (>10years) was a significant individual predictor of ESKD.

Trautmann et al.^17^ in a large cohort of SRNS patients reported a ten-year ESKD-free survival rates of 94, 72, and 43% in cases that achieved complete remission, partial remission, and that showed resistance to intensified immunosuppressive therapy, respectively. Patients who had MCD as histopathology showed better survival (79%) than those with FSGS (52%). Further, PodoNet registry strengthened the evidence that response to initial immunotherapy and underlying genetic mutation are important independent prognostic indicators in addition to the histopathological type, time of diagnosis, age of onset, and kidney function at first presentation in patients with SRNS^17^. In the present study, none of the factors significantly affected the remission status and progression to CKD.

Adverse effects observed were hypertension, cushingoid appearance, short stature, cataract, and obesity in 37.7, 29.8, 25.5, 17.5, and 0.7% of cases, respectively. Inaba et al.^19^ reported hypertension in 31.9%, short stature in 7.2%, and obesity in 5.8% of cases. Ali et al.^24^ reported drug-related complications such as cushingoid appearance (9.2%), hirsutism and gingival hypertrophy (2.3%), and short stature (1.5%). Therefore, attention should also be paid to such adverse-effects during the course of treatment and managed accordingly.

## Mortality

Eight patients (7.3%) died during the study period. Sepsis (3.6%), CKD (2.7%), and COVID-19 (0.9%) were contributors. Mekahli et al.^6^ reported a 3.5% mortality rate due to ESKD. SRNS patients are on long-term immunosuppression and can acquire fatal infection and also progress to CKD. These are important factors responsible for mortality.

The strength of the present study is that we analyzed 110 cases of SRNS in a single-center for their cumulative remission status and kidney function survival over a period of 5 years. The limitations were the short follow up and patient dropout. Therefore, multicenter studies and long-term follow-up of SRNS patients in the Indian population are needed to know their kidney function survival and its predictors, especially in view of the low incidence of genetic mutations in our country.
